# Local understandings of PTSD and complex PTSD among health professionals working with adolescents in violent neighbourhoods of São Paulo city, Brazil

**DOI:** 10.1186/s12888-022-03821-6

**Published:** 2022-03-18

**Authors:** Alessandro Massazza, Juliana Feliciano de Almeida, Meaghen Quinlan-Davidson, Renata Teixeira da Silva, Delanjathan Devakumar, Maria Fernanda Tourinho Peres, Glyn Lewis, Ligia Kiss

**Affiliations:** 1 Department of Health Services Research and Policy, London School of Hygiene and Tropical Medicine, 15-17 Tavistock Pl, London, WC1H 9SH UK; 2 Department of Preventive Medicine, School of Medicine, University of São Paulo, São Paulo, Brazil; 3 Institute for Global Health, University College London, London, UK; 4 São Paulo Municipal Health Department- Medical Residency Program in Psychiatry, São Paulo, Brazil; 5 Division of Psychiatry, University College London, London, UK

**Keywords:** Community violence, Brazil, Adolescents, PTSD, CPTSD, ICD-11, Health professionals

## Abstract

**Background:**

Adolescents in low-resource urban settings in Brazil are often exposed to high levels of trauma that can result in post-traumatic stress disorder (PTSD). However, preliminary evidence indicates that PTSD tends to be under-reported in Brazilian health services, despite the high prevalence of trauma. Additionally, little is known about the perceived applicability among clinicians of the new ICD-11 diagnosis of complex PTSD (CPTSD), despite its potential relevance for contexts of chronic trauma. The current study investigated local understandings of PTSD and CPTSD among health professionals working with adolescents in violent neighbourhoods of São Paulo city.

**Methods:**

Semi-structured interviews were conducted with 58 health professionals working at both the primary care and specialized mental health levels in two areas of São Paulo city with high levels of community violence.

**Results:**

Most participants knew about PTSD, but most did not know about CPTSD. There were mixed views concerning the commonality of PTSD among adolescents exposed to community violence. Many participants reported having no experience working with patients with the PTSD diagnosis. According to some, community violence was normalized by adolescents and health professionals, and did not result in PTSD. Others highlighted how they did not use psychiatric diagnoses in their practice, had critical perspectives towards psychiatric diagnoses and/or PTSD, or simply knew little about PTSD. Furthermore, many highlighted how the chronic nature of multiple traumas experienced by adolescents often resulted in complex clinical presentations characterised by many symptoms beyond PTSD. The diagnosis of CPTSD was considered appropriate to the context by many participants as it captured the complex traumatic histories and symptom presentations of adolescents exposed to community violence in Brazil.

**Conclusions:**

These findings have important implications for the assessment and treatment of mental health among adolescents exposed to community violence in Brazil.

**Supplementary Information:**

The online version contains supplementary material available at 10.1186/s12888-022-03821-6.

Globally, youth violence represents a serious public health concern with an estimated 200,000 homicides occurring among youth 10–29 years of age each year, making it the fourth leading cause of death for people in this age group [1]. Adolescents in low- and middle-income countries (LMICs), and especially in Latin America, are particularly exposed to community violence [2]. Post-traumatic stress disorder (PTSD) is considered one of the most common mental health consequences of exposure to community violence [3]. Nonetheless, there is limited evidence on how health providers from LMICs understand and use this diagnosis in their work with adolescents exposed to high levels of community violence. This represents a significant gap in the literature, especially in light of the criticism advanced regarding the cross-cultural validity of the PTSD diagnosis [4] and the risks of medicalising and depoliticising social suffering through this diagnosis [5].

Adolescents living in low-resource, urban settings in Brazil are exposed to a high number of potentially traumatic events [6]. The prevalence of exposure to traumatic events has been estimated to be as high as 86% in the general population living in Brazilian cities, with urban and community violence reported as the most common types of traumatic exposure [7]. Adolescents are particularly vulnerable to community violence in Brazil [8], with homicide being the leading cause of mortality in this age group [9]. In addition to urban and community violence, Brazilian adolescents are also likely to be exposed to adverse childhood events (ACEs) such as parental separation, emotional neglect, and domestic violence. Research estimates that approximately 85% of Brazilian adolescents experience at least one ACE [10].

Exposure to traumatic events such as community violence among Brazilian adolescents has been associated with increased prevalence of a wide range of mental health problems including substance abuse, depression, and anxiety [11, 12]. It also has also been linked to PTSD [13]. One study reported a relatively high prevalence rate of PTSD (7.8%)^1^ among Brazilian adolescents associated with exposure to violent events such as community and family violence [15].

However, despite the high prevalence of trauma exposure and reported PTSD in epidemiological studies, evidence indicates that PTSD remains largely underdiagnosed within Brazilian healthcare services. For example, one study conducted in Rio de Janeiro highlighted how, despite PTSD being highly prevalent among patients presenting to a mental health outpatient service (20.5% of sample screening positive for PTSD using the Structured Clinical Interview for DSM-IV), it remained undiagnosed by psychiatrists in 97.6% of cases [16]. While particularly high in the current context, under-detection of PTSD is not unique to Brazil [17], with under-detection being recognised as a general issue in both primary [18] and secondary health care [19], especially among adolescents [20]. Various reasons have been suggested to explain why PTSD can go undetected in clinical practice. These include the complexity of the PTSD diagnosis in the current Diagnostic and Statistical Manual of Mental Disorders (DSM-5) [21] as well as the high level of comorbidity between PTSD and various other disorders such as depression [22]. However, to the authors' knowledge, no study has yet investigated possible reasons for the under-detection of PTSD in the Brazilian context, and we are not aware of other studies investigating this issue among health professionals working in other LMICs more generally.

One possible additional reason why PTSD might be under-detected in the Brazilian context is that the type of chronic and repeated traumatic exposure resulting from community and domestic violence might result in a symptom presentation which differs from that of simple PTSD following a one-off traumatic event [23]. Indeed, the most recent version of the International Classification of Diseases (ICD-11) introduced the diagnosis of complex PTSD (CPTSD) to capture disturbances in self-organizations that can sometimes emerge as a result of exposure to multiple, chronic, and repeated traumas from which escape is difficult or impossible [24]. However, except for some psychometric work on a complex PTSD measure [25], no work currently exists on CPTSD in Brazil. No study has yet explored the perceived clinical utility, relevance, and appropriateness of CPTSD among Brazilian healthcare workers. This represents an important research question considering how one of the main goals behind the ICD-11 revisions was to improve both international applicability and clinical utility [26].

To address these gaps in the literature, the current paper aims to answer two main research questions. Firstly, to explore the local understandings of PTSD among health professionals working with adolescents in high-violence neighbourhoods in São Paulo city, Brazil. Secondly, to investigate the perceived clinical utility and appropriateness of the novel diagnosis of CPTSD.

## Methods

### Participants and recruitment

Participants invited were health professionals at five primary health care facilities (Unidade Básica de Saúde, UBS) and two specialised mental health care facilities, including one Child and Adolescent Psychosocial Care Centre (Centros de Atenção Psicossocial Infantojuvenil- CAPS IJ) and one Psychosocial Care Centre for Alcohol and Drugs (CAPS Álcool e Drogas- CAPS AD). Created in 1988, the Brazilian National Health System (Sistema Único de Saúde, referred to as SUS) offers free and universal care to all, with approximately 63% of the Brazilian population in 2015 depending entirely upon its services [27, 28]. We included health professionals from the primary care level as the Brazilian Ministry of Health has recommended this be one of the main entry points to the mental health system [29] and that common mental health disorders should be treated at the primary care level [30].

**Fig. 1:**
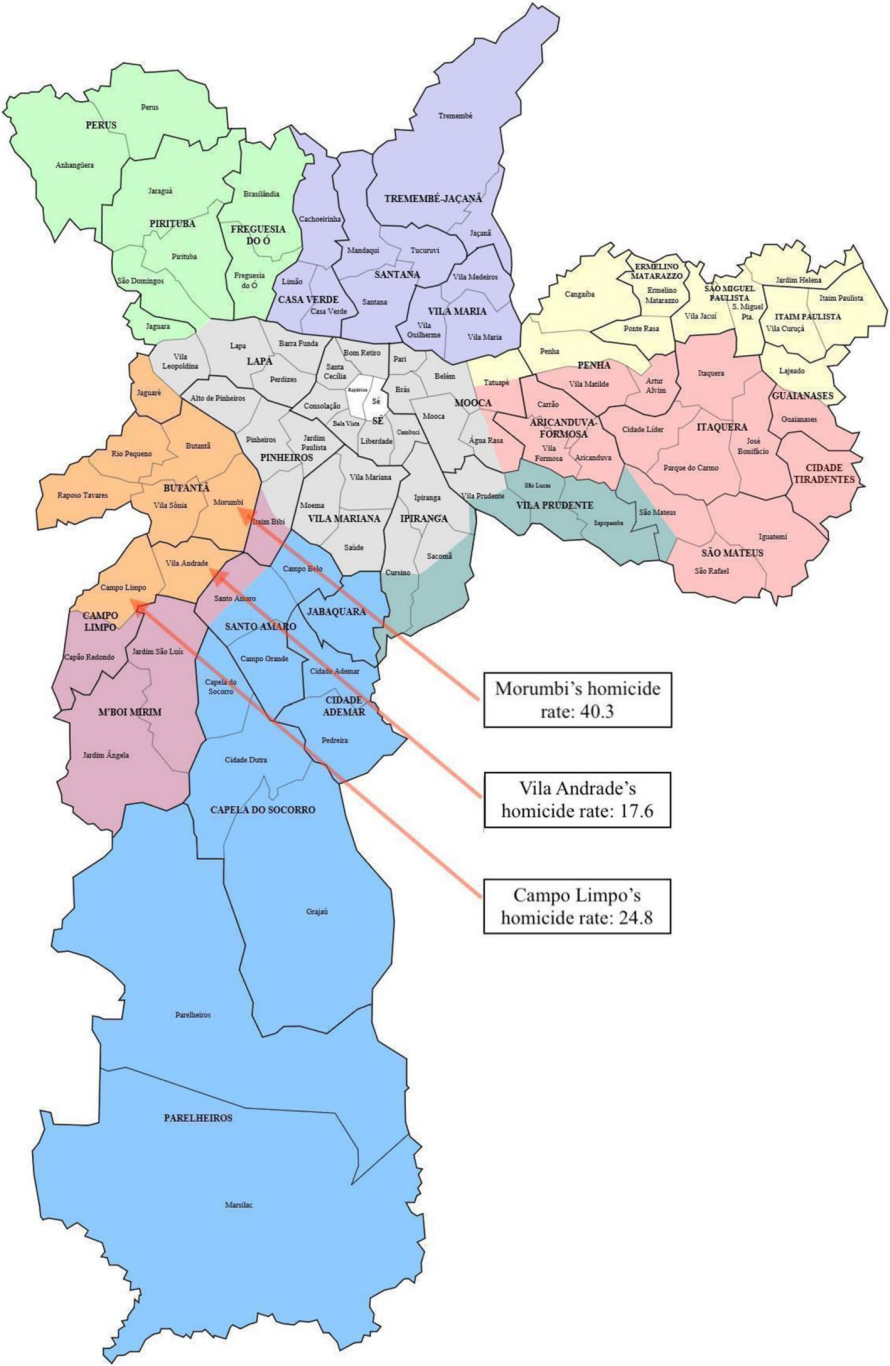
Map of São Paulo’s districts showing the districts in which participants worked with respective youth homicide rates [31]. Note. Map taken from Wikimedia Commons

Participants were sampled from UBS or CAPS clinics in the district of Campo Limpo and in Paraisópolis, a favela in the district of Vila Andrade and adjacent the district of Morumbi. These areas were identified based on their high levels of community violence within the city of São Paulo. In 2021, the youth (15–29 years) homicide rate (value calculated by: total number of deaths due to external cause among people aged 15–29 years old ÷ population aged 15–29 residing in district × 100,000) was estimated at 24.8 in Campo Limpo, 17.6 in Vila Andrade, and 40.3 in Morumbi, compared to the average 16.5 across São Paulo city [31]. In fact, Morumbi had the third highest youth homicide rate in the city in 2021. The location of the different districts within the city of São Paulo is shown below in Fig. 1. Importantly, while average youth homicide rates by district provide an indication of the level of violence in the area, they may under-represent the degree of violence in the contexts where the study was conducted as our participants worked in subareas of these larger districts with higher levels of community violence (e.g., Paraisópolis within the district of Morumbi).

A purposive sampling strategy was used to identify participants through a register from a private health care management company hired by the city of São Paulo and the Brazilian Unified Health System (SUS) to provide public health services (only GPs, psychiatrists, psychologists, nurses, occupational therapists, social workers, and community health workers were selected for participation). Participants were contacted by email or by phone and invited to participate in the study.

## Procedure

Semi-structured interviews were conducted in Portuguese by researchers experienced in qualitative data collection. The interview focused on adolescent mental health and community violence. However, the current paper's focus is on the section covering PTSD and CPTSD (topic guide for this section presented in Additional file 1). The questions on PTSD and CPTSD were left to the end of the interview to assess whether these diagnoses would be mentioned spontaneously during the interview. In the days prior to the interview, the diagnostic definitions of both PTSD and CPTSD as per ICD-11 and the informed consent form were sent via email to the participants (see Additional file 1). In case the participant reported not having read the diagnostic definitions they had received via email (or not remembering them) these were read aloud by the interviewer before the start of the interview section on PTSD/CPTSD.^2^ Interviews lasted approximately one hour each and were video and audio recorded on Zoom, Skype, or WhatsApp. The sample size was determined to cover a range of services and a range of different health professions. Data collection took place during the months of June and October 2020. The study was approved by University College London’s Ethics Committee, the University of São Paulo’s Committee for Ethics in Research and Brazil’s National Research Ethics Committee. Participants who agreed to participate were asked to digitally sign their names and return the informed consent form via email to the interviewer.

## Data analysis

All interviews were transcribed verbatim. Thematic analysis was used to analyse the data [32]. One author (AM) first drafted a preliminary coding framework by reading through the entire dataset. Subsequently, to ensure reliability of the coding framework, a second author (JFDA) also read through the entire dataset and the coding framework was further revised. The remaining authors read a subset of interviews each and commented on the coding framework. The various codes were clustered into thematically related groups of codes to facilitate analysis. One author (JFDA) then proceeded to code 5 interviews using the coding framework. Another author (AM) then also coded these first interviews, and a discussion took place among the authors focusing on discrepancies and conceptual refinement of the codes. The coding framework was then finalised (see Additional file 1). One author (JFDA) then proceeded to code the rest of the dataset using QDA Miner Lite. One other author (AM) oversaw the coding process and regular meetings took place to address any discrepancies in the coding process, to ensure consistency and reliability in the coding, and to interpret the data. Data was analysed in Portuguese and only selected quotes were translated into English.

We analysed only the sections of the interview corresponding to the set of questions on PTSD and CPTSD. However, we also searched the rest of the interview for spontaneous mentions of “trauma”, “post-traumatic stress”, and “PTSD”^3^ to assess whether these terms emerged spontaneously in the interview without the interviewer prompting for them. Sections where these terms were mentioned spontaneously were also coded. Additionally, for each participant, we recorded using categorical variables (“yes”,” no”, “unsure”) whether they had experience working with patients with these diagnoses, whether they believed these diagnoses were appropriate, useful, and common, and whether they had examples of working with patients with such diagnoses. When reporting percentages, we indicate the denominator between brackets to facilitate interpretation considering missing responses. Despite the qualitative nature of this study, we decided to report basic descriptive statistics for context, considering the relatively large sample of participants.

## Results

### Descriptive information on participants

Descriptive information on participants is shown below in Table 1.

**Table 1:** Descriptive information on sample (*N* = 58)

Construct	Percentage or mean
Gender	
Female	78% (*n* = 45)
Male	22% (*n* = 13)
Occupation	
Medical doctor	36% (*n* = 21)
Nurse	22% (*n* = 13)
Social worker	12% (*n* = 7)
Psychologist	9% (*n* = 5)
Occupational therapist	9% (*n* = 5)
Community health worker	7% (*n* = 4)
Other (e.g., physiotherapist)	5% (*n* = 3)
Level	
UBS (primary care)	62% (*n* = 36)
CAPS Infantojuvenil (specialised care)	22% (*n* = 13)
CAPS Álcool e Drogas (specialised care)	16% (*n* = 9)
Area	
Campo Limpo	66% (*n* = 38)
Paraisópolis	34% (*n* = 20)

Note. *UBS* Unidade Básica de Saúde, *CAPS* Centros de Atenção Psicossocial

## Knowledge and perceptions of PTSD and CPTSD

During the interview, only 12% of participants spontaneously discussed trauma or PTSD prior to the questions specific to PTSD and CPTSD, despite the interview focusing on community violence and adolescent mental health. Among participants who were asked whether they knew the diagnosis of PTSD (*n* = 43), most participants (86%) responded that they did. Among participants who were then asked whether they believed PTSD was common among adolescents exposed to community violence (*n* = 40), 63% responded that it was, while 37% believed it was not common. When participants were asked whether they had experience working with patients with this this diagnosis (*n* = 49), half (51%) responded that they did and the other half (49%) that they did not. Among participants who were asked whether they considered the ICD-11 diagnosis to be useful and appropriate for their services (*n* = 25), all considered the diagnosis to be useful and appropriate. Among participants who were asked whether they could describe a real example of a patient with PTSD (*n* = 41), 78% were able to do this.

Conversely, when asked whether they knew the diagnosis of CPTSD (*n* = 34), most participants (78%) reported not knowing about the CPTSD diagnosis. However, when asked whether they believed the ICD-11 diagnostic description was appropriate for the symptom presentation of adolescents exposed to community violence in their practice (*n* = 30), 93% responded that it did. Similarly, when asked whether they could think of an example of a patient that could fulfill the diagnostic criteria for CPTSD because of community violence (*n* = 34), 68% participants could think of an example.

Several recurring themes were identified when analyzing the data that provide more qualitative insight into the data presented above. These themes are described below.

## Habituation and normalization of violence

One possible reason why PTSD was perceived to be uncommon by various participants was that some described a degree of normalization of violence within the community. Participants perceived that both adolescents and health professionals shared this experience. Participants described how violence was “trivialized” and “naturalized” or perceived as part of the “routine” among people living and working in the community. One consequence of the normalization of violence was that participants believed that some adolescents had become habituated to a degree of exposure to community and others forms of violence.

“It’s because I think that violence is so trivialized that it [the violence] doesn’t affect them much. They tell about violence as if it was normal. “Oh, my brother was shot, my brother was arrested”, as if it was a routine thing. Sometimes an injured patient arrives, but it is usually an adult, not an adolescent. We have teenagers who put themselves at risk, but they don’t have much ability to judge they are at risk, there isn’t much suffering related to it [to violence], there are no symptoms because of it”

[P2: Female, Medical doctor, CAPS AD, Campo Limpo]

“The violent stressors are there, but with their reality, they do not understand [it] as such. Do you understand? “Ah, someone passed by, [it was] the people from the BOPE [special police tactical unit] with the police shooting in the air”. If it were in a mansion in Morumbi [wealthy district neighboring Paraisópolis] this would create a problem, but it is in Paraisópolis, it happens every week”

[P37: Medical doctor, Male, UBS, Paraisópolis]

According to some participants, this habituation could result in some adolescents experiencing only a blunted psychological response to community violence, rather than resulting in them developing PTSD. The fact that community violence was so pervasive and common in the lives of the adolescents presenting to these services meant that it had somewhat lost its traumatic potential as it had become so integrated within the daily life of the community. One participant described how because community violence was so common, the link to PTSD was only made in the case of extreme types of traumatic events, such as in the case of an adolescent who had been subjected to torture. As highlighted by the quote below, this led some participants to believe that community violence could not be a trigger for PTSD.

“We do not recognize community violence as a trigger for PTSD. We don’t keep doing that [not recognizing community violence]. We recognize more sexual violence as a trigger for PTSD, more in this sense. […] I also think that we are in such a precarious territory that we also end up normalizing the different situations of violence. And then community violence ends up being normalized”

[P4: Psychologist, Female, CAPS IJ, Campo Limpo].

Importantly, not every participant believed that adolescents could simply habituate to daily exposures of trauma and community violence. On the contrary, several highlighted the profoundly detrimental impacts that growing up as an adolescent in highly violent contexts could have on mental health. While violence might have been normalized, it could nonetheless lead to deep psychological suffering.

“Any running around, and one is already scared. If anyone is running, it is already a reason to be thinking… if you hear fireworks, you already think it’s a gunshot. Thus, there is a high level of stress like that. We receive adolescents who were born there in the community, who were raised there all the time, they even develop schizophrenia, thinking that they are being listened to, that they are being watched, more persecutory [beliefs]. I believe that that is because of the routine [violence] that they end being exposed to”

[P40: Medical doctor, Female, UBS, Campo Limpo]

## Barriers to making the PTSD diagnosis

Whilst several participants perceived PTSD to be common among adolescents exposed to community violence, there was a consensus that few adolescents ended up receiving a PTSD diagnosis. This was reflected by the fact that, among participants who knew the PTSD diagnosis and who were asked if they had experience working with patients with a PTSD diagnosis, only half responded that they did. Furthermore, even among participants who reported having experience working with the diagnosis, this experience was often limited to only a few cases.

“Interviewer: Right. And do you consider that the diagnosis of PTSD is common in cases of adolescents who are exposed to community-based violence?

Participant: Yes. It's common.

Interviewer: And have you already made this diagnosis Dr. [name of participant]?

Participant: No. Not like that. But it is common”

[P39: Medical doctor, Female, UBS, Campo Limpo].

“It is very rare for teenagers to be diagnosed, but there are many teenagers out there, in the community, at home, with this diagnosis [PTSD] without knowing, sick without proper support"

[P18: Social worker, Female, CAPS AD, Campo Limpo].

“I never did it [PTSD diagnosis]. And it's weird because we really live in a very violent environment. But I never made this diagnosis"

[P24: Medical doctor, Female, UBS, Paraisópolis].

Several different factors were likely responsible for this. One reason is that certain categories of interviewed participants such as psychologists or nurses did not perceive themselves as responsible for making psychiatric diagnosis using ICD or DSM criteria.^4^ Participants who were not medical doctors, highlighted how they generally did not rely on diagnostic systems (whether based on ICD-11 or DSM-5) but used alternative taxonomies that were specific to their field. For example, one nurse described how, in her own work, she relied on “nursing taxonomies” [taxonomias de enfermagem]. Similarly, psychologists often described focusing more on the symptom, on the presentation of the patient, and on the formulation of the mental health problem, and less on specific diagnostic categories.

“We have other diagnoses, we work, of course, with nursing diagnoses that are focused on the process of caring for the person and the community”

[P33: Nurse, Male, UBS, Paraisópolis].

“I'll have another perspective that isn't the diagnosis, [my perspective] I think it's more about the complaints/symptoms [queixa], when they bring the complaint/symptom [queixa], the team is also like that [also has this perspective]. We use the medical diagnoses here, we do, but we work on what the teenager brings, how they present himself, putting themself in the world”

[P8: Psychologist, Female, CAPS IJ, Campo Limpo]

Another possible factor that could explain not using the PTSD diagnosis is some participants expressed critical perspectives towards the construct of psychiatric diagnoses in general and of the PTSD diagnosis specifically.

“I think [of PTSD] like many other diagnoses. I keep thinking that diagnosis is an important thing, diagnosis is important, but it cannot be the basis of care, so that we do not label the other as a diagnosis. Diagnosis is one more thing in a person's life”

[P22: Psychologist, Female, CAPS AD, Campo Limpo]

“I have a more descriptive perspective around diagnoses, and less of the ICD or DSM diagnoses. Not that I'm against it, I think it's a symbol, that it has a narrative and when you talk about it it's clear what you mean. But my outlook is an outlook that is more focused on the description. […] The diagnosis of post-traumatic stress, it tells us about an experience that stood out in that teenager's life compared to others. And there is an experience that brought a consequence or some consequences that we need to look at, but it is also one [experience], it is delicate because it is still one experience within other experiences of life. So, we look at that [experience], but we have to be very careful not to think that it is just, that it is exclusively that, that it is essentially that. So, an episode of sexual violence can generate a disorder, a PTSD, but there is a whole context around it. There is a whole history, there is a whole family, a society, we look at the episode of violence, not forgetting to look at others [factors]. So, it's important, but we have to use it and look [at it] very carefully"

[P9: Psychologist, Male, CAPS IJ, Campo Limpo].

This more critical perspective was often rooted within narratives that were aligned with a social medicine approach and that placed the emphasis on the sociopolitical and structural determinants of mental health rather than on diagnosis at the individual level. Participants described how mental health was something that emerged from the context in which adolescents lived rather than in the isolation of their minds. Mental health did not exist in a vacuum but was inextricable from social, economic, and political circumstances. As a result, the nexus between trauma and PTSD always had to be seen through the prism of context.

“The problem is not just post-traumatic stress disorder, but the whole environment. It is not enough for us to treat PTSD; we also have to make the other approaches and other appropriate referrals"

[P49: Medical doctor, Male, UBS, Campo Limpo].

The PTSD diagnosis was at times perceived by some participants as incapable of capturing the ongoing nature of the stressors to which participants were exposed, as there was no “post-trauma” in the lives of the adolescents living in these communities. Additionally, the focus on trauma was perceived by some as insufficient in the context of ongoing structural difficulties. The mental health problems faced by adolescents, rather than resulting from a disorder of memory, were often described as emerging from a deeply disordered present which had to be confronted daily.

“The adolescent's mental health illness has more to do with these structural issues than a traumatic one. It's not a war, it's not something that comes out of nowhere, it's a structural and transgenerational thing, I think illness has more to do with this than with trauma. So, you can have, and there are, I have cases […] where violence manifests itself as trauma, it's not that not, I think violence has traumatic potential. But overall, […] my experience is prevalently more structural than traumatic. […] Yes, I agree with that, at least most of the suffering I deal with is not due to trauma, but because this violence is already experienced in a structural way within early childhood"

[P26: Psychologist, Female, UBS, Paraisópolis].

This perspective often translated into specific ideas about appropriate treatment for adolescents exposed to community violence. Certain participants expressed the perception that providing a PTSD diagnosis was of only partial utility if it could not lead to addressing the root causes of the distress experienced by the adolescents. For these participants, the appropriate treatment for the complex mental health needs of adolescents exposed to violence went beyond making the appropriate diagnosis, but had to be holistic in nature, characterized by complex interactions between a variety of different health, social, and judicial services.

"How am I going to attend someone who has gone through a post-traumatic disorder if I'm not going to support them all the time, a quality service with knowledge, with a qualified professional in the time that it needs, as often as it needs, giving support for their family, because the trauma is not just of the adolescent, often the adolescent's story is the story of the house, so the disease is much further back, the situation is much more complex [lit. the hole is much lower]"

[P44: Medical doctor, Female, UBS, Campo Limpo].

"If I identify this teenager right after the trauma, what we know is that the treatment there, at first, is support, and not necessarily for me to bring this teenager to talk about what happened, but I can assess whether they remain exposed to a situation of violence, to take the measures I need to take to get them out of the situation of violence, without provoking another violence. I'll try to get them away from this situation of violence, bond with this teenager, show them that we are a partner service and available to them, someone they can ask for help if they need it, involve them frequently. […] It is more a work of bonding, monitoring and support, [rather] than treating symptoms, giving the diagnosis itself. It's protection"

[P7: Medical doctor, Female, CAPS IJ, Campo Limpo]

Importantly, while some participants expressed critical views around the diagnosis of PTSD, other participants highlighted the importance and the appropriateness of the diagnosis for the context in which they worked. Therefore, views around the utility and value of PTSD were mixed in the current sample.

“In post-traumatic disorders, I believe that, as a diagnosis, it is essential for the management of the case. Yes, I think it is a valid diagnosis, yes"

[P17: Nurse, Female, CAPS AD, Campo Limpo].

“Interviewer: And do you think it's useful, applicable to your service [the PTSD diagnosis]?

Participant: Of course, yes, yes. Even now, in a very short time, something like 10 days, we don't get adolescents, but we care for victims of rape, so it does, post-traumatic disorder, the diagnosis is important”

[P34: Nurse, Male, UBS, Paraisópolis].

One final factor that might have contributed to some participants not working with the PTSD diagnosis was a perceived lack of precise knowledge around this disorder. This was more present among participants working in primary health care, some of which had never even heard of the PTSD diagnosis. Many other participants working in primary health care reported “having heard” of the diagnosis but not having in-depth knowledge about it. Some participants considered this to be the result of a lack of training on the disorder and on appropriate treatment options.

“Interviewer: Do you know the diagnosis of Post-Traumatic Stress Disorder?

Participant: I've heard about it, but I don't, I don't have deep knowledge, I don't know it very well"

[P53: Nurse, Female, UBS, Campo Limpo].

"I think we don't have training to be able to give a correct [PTSD] diagnosis or correct approach, it's not just diagnosis, it's a post-diagnosis approach, I think we don't have training for this”

[P44: Medical doctor, Female, UBS, Campo Limpo]

This perceived lack of knowledge around the diagnosis of PTSD among workers in primary health care was often related to the idea that PTSD was a diagnosis that required specialized mental health knowledge and that was not within the remit of the UBS. This was reflected in the habit of referring cases of probable PTSD identified in the UBS to CAPS. The perceived lack of knowledge about PTSD in primary health care was also reflected in a perceived lack of knowledge about the appropriate treatment for PTSD. Indeed, for some participants the perceived limited utility of the PTSD diagnosis was the result of not knowing what a diagnosis of PTSD would have meant for treatment and care.

“I don't know to what extent knowledge about the diagnosis, of a diagnostic hypothesis, would change, at least my way of caring”

[P29: Occupational therapist, Female, UBS, Paraisópolis].

## Trauma and mental health beyond PTSD

Another theme identified among participants was the perception that PTSD was not the only, or the most common, mental health consequence experienced by adolescents exposed to community violence. On the contrary, when asked to provide an example of a case of PTSD resulting from community violence, the symptoms described often eluded a classic PTSD presentation but included a very heterogeneous range of symptoms. These included symptoms of depression and anxiety, self-harm and suicide, substance misuse, behavioral and relationship problems, sleep disturbances, and other symptoms such as psychosis and psychosomatic complaints. Quotes by participants describing the different symptoms resulting from exposure to trauma and community violence are provided in Additional file 1.

“Interviewer: And do you consider that the diagnosis of PTSD is common in cases of adolescents exposed to community-based violence?

Participant: So, I see that there is some, but I don't know if we give this diagnosis so much, I see more that it [community violence] generates depression, it generates anxiety, it generates fear. But regarding this diagnosis, I don't know if we have that many [cases]. But it does happen"

[P11: Social worker, Female, CAPS IJ, Campo Limpo].

For some participants, PTSD-like symptoms were a key characteristic of the response to community violence. These participants often described adolescents with re-experiencing symptoms such as intrusive memories, flashbacks, and nightmares as well as avoidance symptoms such as not wanting to leave one’s home after having been exposed to community violence.

"Adolescents who experience violence, they tend to develop some kind of disorder, and post-traumatic disorder is the most common"

[P51: Medical doctor, Male, UBS, Campo Limpo].

However, PTSD-like symptoms were rarely described in isolation and a transdiagnostic understanding of trauma and mental health was common among participants. Comorbidity between different types of mental health problems was considered the norm in the adolescent population presenting to the services, with participants describing the typical patient as a “combo” of diagnoses. While trauma was generally thought to leave “marks” [marcas] on adolescents, these marks were not clearly associated with a diagnosis of PTSD but covered a broad range of symptoms.

“She started to have a lot of autonomic symptoms, flashbacks, nightmares, she started to avoid going to school, she had a lot of anxious crises at school, and she started getting very depressed too, she had a suicide attempt"

[P6: Medical doctor, Female, CAPS IJ, Campo Limpo]

The boundaries between different conditions were often not clearly demarcated with symptoms evading diagnostic constructs and interacting in complex ways and reinforcing one another. The idea of a single diagnosis with neat boundaries was not reflected in a messy clinical reality of complex, comorbid presentations.

“A boy who was probably sexually abused before he was 4 years old, and he is a boy who came to us with very different symptoms. It was not possible for us to make a specific diagnosis for him. He looked like a boy who was developing a personality disorder there, but he had a lot of risky behavior, self-harm, fugue states [fuga], he complained of psychotic symptoms, he had a very high demand for care, a lot of difficulty in bonding with others, a lot of self-aggression and little response to drug treatment like this"

[P7: Medical doctor, Female, CAPS IJ, Campo Limpo]

The complexity inherent to symptom presentations was compounded by the complex nature of the traumatic exposure adolescents had to live through. Adolescents described by participants had often been exposed to a multitude of different chronic traumatic exposures rather than to a single traumatic event. While the interview focused mainly on community violence, it was often difficult for participants not to touch on the various other types of violence endured by the adolescents. These included violence at home within the family (often of sexual nature), violence within school settings such as bullying by other students, and violence from the State (e.g., house raids by the police). These different types of violence also interacted with one another and reinforced each other in complex ways. For example, one participant described the case of a mother burning her son with an iron as she believed that punishing him would have been the only way to prevent him from getting involved in gangs and community violence.

"I've seen a mother who burned her son's hand with an iron because she had no other way, she thought, "Wow, I live in a place that has a lot of criminals, if I don't give a very serious punishment, he'll be a criminal"

[P2: Medical doctor, Female, CAPS AD, Campo Limpo].

**Fig. 2:**
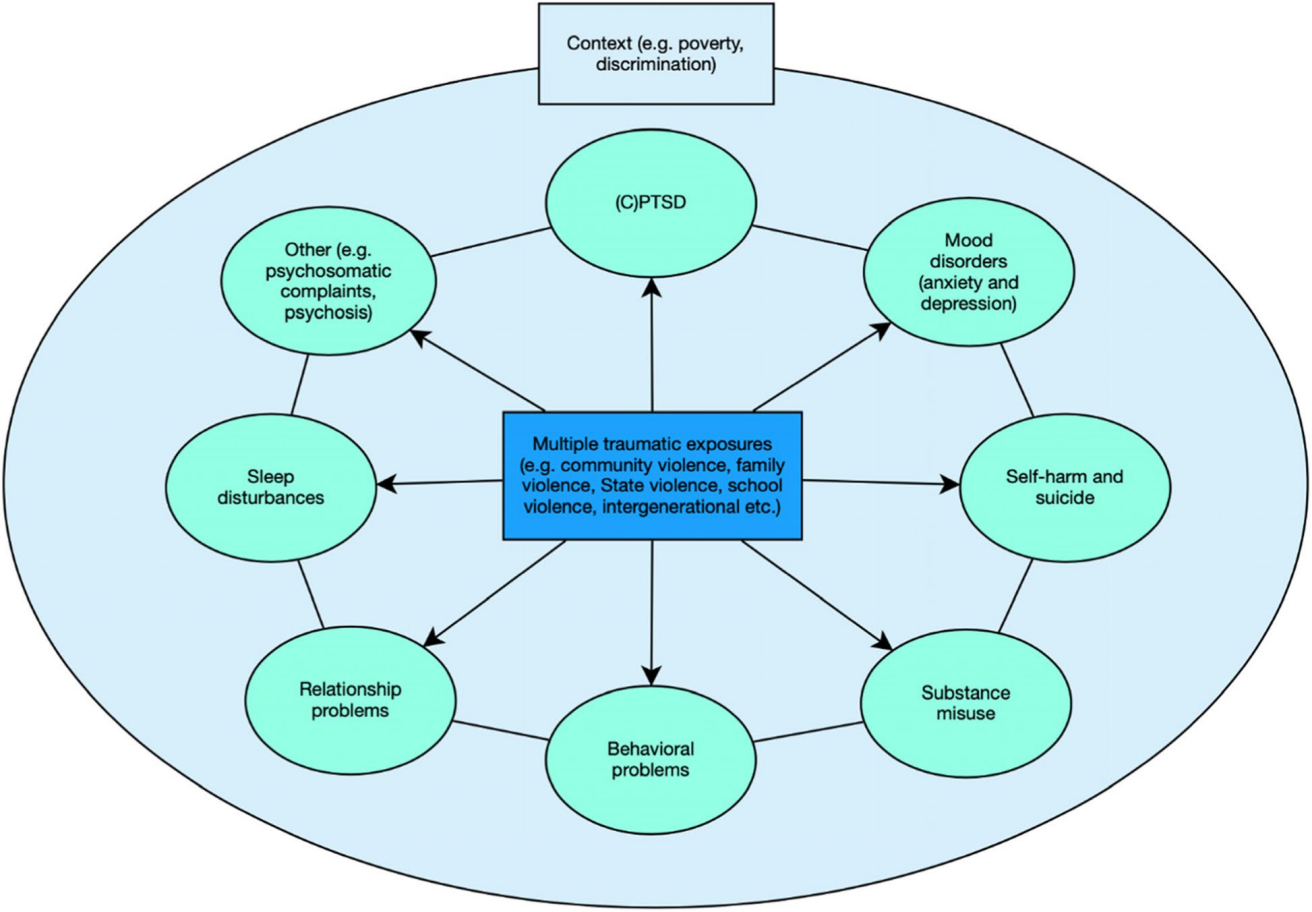
Relationship between trauma and mental health as described by local health workers

Therefore, complex trauma histories translated themselves into complex clinical presentations, which were made even more complex by a background context characterized by poverty, deprivation, intergenerational transmission of trauma, and discrimination. This is summarized visually in Fig. 2 below.

The complex and comorbid nature of the mental health problems experienced by the adolescents meant that PTSD was at times misdiagnosed, clustered under other disorders with which the clinician had more familiarity such as anxiety, or simply missed. Another consequence was that in the context of limited resources, clinicians often had to attend to symptoms which were perceived as being more severe or requiring more urgent attention (e.g., self-harm or suicidal ideation), meaning that PTSD was put aside.

“Interviewer: Right. And have you already made this diagnosis [PTSD]?

Participant: No. Maybe I have failed to observe it [passado batido], but I do not remember. It [PTSD] ends up being fitted into a little house of anxiety and depression"

[P38: Medical doctor, Female, UBS, Paraisópolis].

"But post-traumatic stress disorder comes a lot to us, it's just that it's not noticed at first"

[P23: Medical doctor, Female, UBS, Paraisópolis].

"This [PTSD] is very common, but we do not give the diagnosis, we understand the issue, we understand that this issue is a determining issue of mental health and treat the anxiety disorder, usually in children, it is an anxiety disorder […]. So, our reading, at least mine due to lack of knowledge, does not think that the child has post-traumatic stress disorder, we treat the symptomatology, let’s say"

[P3: Medical doctor, Male, CAPS IJ, Campo Limpo].

## CPTSD as appropriate to adolescents exposed to community violence

As shown in the descriptive section above, most participants had never heard of the diagnosis of CPTSD prior to the interview or had some misunderstandings about the diagnosis (e.g., thinking that CPTSD captured PTSD with other comorbid mental health conditions).

“Interviewer: Do you know the diagnosis of Complex Post-Traumatic Stress Disorder?

Participant: Not complex, I don't know what this diagnosis is"

[P35: Medical doctor, Female, UBS, Paraisópolis].

However, after having read the ICD-11 description of CPTSD, most participants highlighted how they perceived the diagnosis to be particularly appropriate for the symptom presentation of adolescents exposed to community violence. Participants highlighted how the description of the stressors as “series of events of an extremely threatening or horrific nature, most commonly prolonged or repetitive events from which escape is difficult or impossible” resonated with the type of traumatic exposure that was common among adolescents presenting to their services. As highlighted above, one of the critiques posed to the PTSD diagnosis was that this diagnosis appeared to assume that a situation of “post-trauma” existed in the lives of the adolescents, which was rarely the case in the community. On the contrary, CPTSD was able to capture the chronic and repeated nature of the exposure to different types of violence endured by adolescents.

"I think it [CPTSD] makes a lot more sense than the diagnosis of classic PTSD”

[P7: Medical doctor, Female, CAPS IJ, Campo Limpo].

“What I read about post-traumatic stress is more or less like this: “Oh! That acute event, let's say a robbery, a rape and that person has it [PTSD]”, there is that would be what I understood about post-traumatic stress. From the moment you have complex post-traumatic stress disorder, which are repetitive actions of intense suffering, physical and emotional violence, this is very common, and we do not give this diagnosis and I believe that this is crucial for mental health. This [CPTSD] is very common in situations that live with vulnerability, whether at home or at school, gender issues, sexuality, this is very common, we notice [it] a lot, we don't use this name “complex post-traumatic stress disorder”, but we see a lot [of it] here”

[P3: Medical doctor, Male, CAPS IJ, Campo Limpo]

As highlighted in Fig. 2, the norm for adolescents was to be exposed to recurring, chronic, and to different types of violence rather than one-off acute traumatic events. Similarly, adolescents could not escape from the exposure as trauma took place in the very same places where they lived and spent most of their time, within schools, homes, and communities. As one participant put it: “these people cannot get out of this cycle, they remain inside this stressful environment”.

"These issues, for example, related to prolonged domestic violence and sexual abuse are quite common"

[P46: Occupational therapist, Female, UBS, Campo Limpo].

Some participants also highlighted how the additional symptoms in CPTSD (i.e., affect dysregulation, negative self-concept, and disturbance in relationships) contributed to capturing the more complex clinical presentations that were typical of the adolescents presenting to their services and which PTSD did not cover.

“Look, when they [teenagers] come to us, I think they've passed PTSD like that, I think they've already developed some major disorder. [...] Here, as they are often exposed to violence from an early age, they grow up in the community in a context of violence, they do not arrive with PTSD for us, they already arrive with some other more serious disorder"

[P19: Social worker, Female, CAPS AD, Campo Limpo].

“Because it is from this history of chronic violence that he had at home that he was like this. Anxious, with terrible self-esteem. Then this week he was saying that he is starting to lose his relationship, because of this insecurity, of thinking that he doesn't deserve the other's love. So, I think it [CPTSD] applies. This is just an example, but I think it [CPTSD] applies”

[P16: Nurse, Female, CAPS IJ, Campo Limpo]

As shown in the quote above, although most participants did not know about the CPTSD diagnosis prior to the interview, many were able to retrospectively think about cases that fit the diagnostic criteria described in the ICD-11 description.

"This patient, I even think he [has] complex [PTSD]"

[P42: Community health worker, Female, UBS, Campo Limpo].

“But the guilt and the shame are so great because the mother put her finger in her face, “you were the one to blame for him doing that, because you are a lecher and such”. So her guilt was so great, that the guilt was her own imprisonment, imprisonment. So, I think it's Complex [PTSD]"

[P23: Medical doctor, Female, UBS, Paraisópolis]

"There's an adolescent of [that had experienced] recurrent sexual violence with little family listening, so when the person spoke to the family, they didn't believe [her], she has a lot of difficulty in having relationship with people, more long-term relationships, so her affections are very oscillating and at all times she has a feeling of guilt she has, of self-deprecation. I think it fits into this Complex PTSD"

[P13: Occupational therapist, Female, CAPS IJ, Campo Limpo]

## Discussion

The current study represents the first attempt to investigate the local understanding of PTSD and CPTSD among health professionals working with adolescents in São Paulo city’s neighbourhoods characterised by high levels of community violence. In the current sample, most participants knew about PTSD, but most were still unfamiliar with the new ICD-11 diagnosis of CPTSD. Participants held mixed opinions concerning the perceived commonality of PTSD and many did not have direct experience working with patients with a PTSD diagnosis. This provides support to the findings around the possible under-reporting of PTSD in the Brazilian health system [16].

The lack of experience working with the PTSD diagnosis could be explained by different factors. According to some participants, because community violence was so common, it ended up being normalized and some adolescents habituated to it and did not develop PTSD. Studies from other contexts affected by chronic trauma exposure have suggested that under certain conditions some people might display a degree of habituation and resilience to recurring violence [33, 34]. However, this finding should be interpreted cautiously for several reasons. Firstly, the perceived habituation described by some participants in our sample is in contrast with the high rates of PTSD identified in epidemiological studies among communities exposed to high levels of community violence in Brazil [7, 35], including among adolescents [15]. Secondly, this was an opinion expressed by some health professionals and might not reflect the lived experience of the adolescents exposed to community violence themselves. Thirdly, this perspective was not shared among all health professionals with many participants highlighting the detrimental consequences that community violence had on adolescents’ mental health. Finally, while according to some participants community violence did not result in specific psychiatric disorders such as PTSD, it could still cause substantial levels of suffering. Future studies might investigate more systematically the degree of habituation in settings exposed to chronic violence in Brazil and investigate its possible relationship with mental health.

Some participants also reported not working with ICD/DSM diagnoses in their practice (e.g., psychologists, nurses) and/or having critical perspectives towards the notion of psychiatric diagnoses in general. Many of these participants expressed opinions that aligned with a social medicine approach which focused more on social suffering and on understanding the role of social, structural, and political determinants in producing mental health and illness. This perspective was in contrast with a more rigid biomedical approach which centred on specific diagnostic categories at the individual level. Additionally, some participants expressed perspectives that aligned more with critical psychiatry stance and the anti-asylum movement [movimento antimanicomial] in Brazil [36]. Finally, some participants reported preferring to focus on treating problematic symptoms, independently from whether they were part of a specific disorder such as PTSD; an approach that is particularly common in clinical psychology but that is also present in psychiatry [37].

This finding needs to be contextualized within a historical tradition of social medicine and collective health in the Brazilian health system rooted in principles of social justice [38] and in the psychiatric reform that took place in the late 1980s and that aligned with a social medicine approach [39]. For example, guidelines from the Ministry of Health for the basic care of mental health problems among children and teenagers make little reference to specific psychiatric diagnosis with the focus being on social suffering [sofrimento social] [40].^5^ Future studies could investigate more specifically how mental health professionals in Brazil navigate the possible tensions between a social medicine approach to health and illness and the use of mental health diagnostic categories. One possible practical implication of this finding would be that of working towards an improved integration of both biomedical and psychosocial perspectives within national clinical guidelines, as per the biopsychosocial model [41]. This would ensure that patients experiencing PTSD symptoms in similar high-violence contexts receive appropriate care at the individual level while simultaneously recognising the social-political nature of the roots of their suffering.

Many participants also highlighted how the chronic exposure to multiple traumas experienced by adolescents resulted in a complex array of symptoms that often went beyond simple PTSD (see Fig. 2 and Additional file 1). This finding is in line with research showing how exposure to trauma in general as well as community violence specifically is likely to result in several mental health problems beyond PTSD among adolescents, such as depression [42], anxiety [43], substance abuse [44], and self-harm [45], with high-levels of comorbidity between PTSD and other disorders [46]. Indeed, participants highlighted how comorbid and complex cases were the norm in their adolescent population. One practical implication of this finding concerns the possibility of training health professionals in Brazil in the use of transdiagnostic, brief, and scalable psychological interventions, such as WHO Early Adolescent Skills for Emotions, that have been shown to be effective in addressing an array of symptoms in other contexts affected by adversity [47].

This also represents the first study to assess perceptions of the new ICD-11 diagnosis of CPTSD among health workers in Brazil. Very few participants were familiar with the construct. This was to be expected given that community violence is not included among the examples of traumatic events following which CPTSD may ensue in the ICD-11 description and given how the ICD-11 is a technical document that has been updated only recently, meaning it may take some time for recent additions to trickle down to health professionals. However, the diagnosis was perceived as being appropriate in capturing the symptom presentations of adolescents exposed to community violence and many participants could think about cases in their practice that would have qualified for the diagnosis. Additionally, participants highlighted how the mention of “prolonged and repetitive [traumatic] events” reflected more accurately the settings in which they worked in, characterized by multiple chronic traumatic exposures rather than one-off acute events. Since improvement in international applicability and clinical utility of diagnoses was a core goal behind the ICD-11 revisions (Khoury et al., 2017), this represents an important finding. Based on these findings, an important future research priority will be to assess the prevalence of CPTSD following exposure to community violence in Brazil as well as in other countries with high levels of community violence in the region. Additionally, as the diagnosis of CPTSD becomes more recognised, an additional future research questions concerns whether and how it will be used in clinical practice by health professionals working in settings characterised by elevated rates of complex trauma [48].

A final finding from our study concerns the lack of precise knowledge around the diagnosis of PTSD by some participants, especially in the context of primary health care. This, at times, resulted in PTSD being missed or misdiagnosed. Although this finding should be interpreted cautiously as our sample might not be representative of the general health workforce in Brazil, it does provide a possible explanation for the under-diagnosis of PTSD in mental health outpatient settings in Brazil reported in some studies [16]. Since appropriate diagnosis of PTSD can lead to the patient receiving evidence-based treatment promptly, it is essential that health professionals working with populations with high trauma exposure are familiar with the basics of PTSD assessment and management. The mhGAP Humanitarian Intervention Guide developed by the World Health Organization contains a module specific to the clinical management of PTSD and could represent a useful training resource for primary healthcare professionals working in high-violence neighbourhoods in Brazil. Given the substantial underfunding and degree of excess demand that characterises the Brazilian primary health care system [49], any such specialized mental health training should carefully consider issues of cost and time effectiveness.

The current study has some limitations. Firstly, we only recruited participants from two districts in the city of São Paulo meaning that our findings might not apply to other cities in Brazil or possibly other settings with high levels of community violence. However, given that community violence is perceived as a regional problem in Latin America with several common dynamics across countries [50], our findings might be relevant to other settings in the region. Another limitation is that we only focused on health professionals, and we did not include interviews with the adolescents themselves. The perception of adolescents exposed to community violence around the diagnoses of PTSD and CPTSD would constitute an important separate question for future research. Furthermore, we did not look at gender differences in our sample and future studies might want to explore possible differential perspectives between male and female healthcare providers. Additionally, levels of understanding as expressed in an interview may suffer from self-report issues and future studies may use different methods (e.g., observations, vignettes, or quantitative methods) to tap into different degrees and types of understanding of these diagnoses (e.g., theoretical versus practical understanding) [51]. Finally, because of the semi-structured, open nature of the qualitative interview, the topics covered varied across interviews, resulting in missing data in the descriptive statistics.

Exposure to community violence is common among adolescents living in low-resource urban settings in Brazil and can result in substantial mental health consequences, including PTSD. This represents the first study to qualitatively investigate the local understandings of the diagnoses of PTSD and CPTSD among health professionals working with adolescents in high-violence neighbourhoods in São Paulo. An improved understanding of how clinicians navigate these diagnoses can allow for the identification of clinical blind-spots and areas for improvement in assessment and treatment which in turn will lead to improved adolescent mental health care in the context of community violence.

## Supplementary Information

**Additional file 1.**

## Data Availability

The data will be not publicly available due to information that may compromise the privacy of research participants.

## Footnotes

1 Compared to a 5% prevalence rate among US adolescents [14]

2 While this might have influenced the responses of participants, it was deemed necessary to ensure participants responded to the interview questions with a shared understanding of the constructs under investigation.

3 The Portuguese words “trauma”, "estresse pós-traumático", and "TEPT” were used.

4 Within the Brazilian health system, formal mental health diagnoses are generally the remit of doctors rather than nurses and psychologists (including for legal reasons).

5 Post-traumatic stress disorder (TEPT in Portuguese) is never mentioned in these guidelines (although there are mentions of post-traumatic stress, “estresse pós-traumático”).
