# Supplementary material for: Local understandings of PTSD and complex PTSD among health professionals working with adolescents in violent neighbourhoods of São Paulo city, Brazil
Source: BMC Psychiatry. 2022 Mar 18;22:196. doi: 10.1186/s12888-022-03821-6 (PMC8932201; doi:10.1186/s12888-022-03821-6)
Supplement: Supplementary file 1 — Additional file 1. [file 12888_2022_3821_MOESM1_ESM.docx]

**Appendix 1: Translated topic guide for interview section on PTSD and CPTSD**

1. Do you know about the Post-Traumatic Stress Disorder diagnosis?

Now I would like to present you with the definition of PTSD

[Definition of PTSD as per ICD-11 presented, see Appendix 2]

1.1. [IF YES] Do you consider PTSD common among adolescents exposed to community-based violence? Have you made this diagnosis?

- [IF YES, ASK] Could you give us a real-life example of a case where you diagnosed PTSD due to community violence?

1.2. [IF NO] Is PTSD useful and applicable within your service for adolescents with mental health disorders? Can you think of adolescent service users who would meet this criterion?

- [IF YES, ASK] Could you give us a real example?

2. Do you know about the Complex Posttraumatic Stress Disorder (CPTSD) diagnosis?

Now I would like to present you with the definition of CPTSD

[Definition of CPTSD as per ICD-11 presented, see Appendix 2]

2.1 Do you consider CPTSD appropriate for the psychological presentation of adolescents exposed to community-based violence?

- [IF YES, ASK]: Is this applicable to your service? Could you give us a real example of a case where you diagnosed CPTSD due to community violence?

**Appendix 2: Definitions of PTSD and CPTSD as per ICD-11**

PTSD: <https://icd.who.int/browse11/l-m/en#/http%3a%2f%2fid.who.int%2ficd%2fentity%2f2070699808>

**Description**

Post-traumatic stress disorder (PTSD) is a disorder that may develop following exposure to an extremely threatening or horrific event or series of events. It is characterized by all of the following: 1) re-experiencing the traumatic event or events in the present in the form of vivid intrusive memories, flashbacks, or nightmares. These are typically accompanied by strong or overwhelming emotions, particularly fear or horror, and strong physical sensations; 2) avoidance of thoughts and memories of the event or events, or avoidance of activities, situations, or people reminiscent of the event or events; and 3) persistent perceptions of heightened current threat, for example as indicated by hypervigilance or an enhanced startle reaction to stimuli such as unexpected noises. The symptoms persist for at least several weeks and cause significant impairment in personal, family, social, educational, occupational or other important areas of functioning.

CPTSD: <https://icd.who.int/browse11/l-m/en#/http%3a%2f%2fid.who.int%2ficd%2fentity%2f585833559>

**Description**

Complex post-traumatic stress disorder (Complex PTSD) is a disorder that may develop following exposure to an event or series of events of an extremely threatening or horrific nature, most commonly prolonged or repetitive events from which escape is difficult or impossible (e.g., torture, slavery, genocide campaigns, prolonged domestic violence, repeated childhood sexual or physical abuse). All diagnostic requirements for PTSD are met. In addition, Complex PTSD is characterized by severe and persistent 1) problems in affect regulation; 2) beliefs about oneself as diminished, defeated or worthless, accompanied by feelings of shame, guilt or failure related to the traumatic event; and 3) difficulties in sustaining relationships and in feeling close to others. These symptoms cause significant impairment in personal, family, social, educational, occupational or other important areas of functioning

**Appendix 3: Coding framework**

**Coding framework PTSD and CPTSD**

| **Codes** | **Definition** | **Example** |
| --- | --- | --- |
| **Types of violence** | | |
| Family violence | Descriptions of violence (including sexual violence) perpetrated by parents, family members etc. | “She only started to respond better [to treatment] when she opened about how she had been abused by a family member a while back” [P7] |
| Community violence | Description of violence (including rape) by other members of the community (e.g., gang violence, violence by neighbors) | “I heard of cases in the community where teenagers had family members murdered by the parallel power [popular expression to designate the existence of armed forces parallel to the State such as militia or criminal factions linked to drug trade organizations such as the PCC]” [P34] |
| State violence and interactions with police | Violence perpetrated by State agents (e.g., police or violence within State institutions), arrest | “I think sexual abuse is something that causes a great deal of trouble, police violence, I think that too” [P51] |
| School violence | Violence perpetrated within the school (e.g., by teachers, by schoolmates, bullying) | “At school they are bullied” [P3] |
| Sexual violence | When sexual violence is discussed but not clear by whom it is perpetrated  When it is clear you can code this theme + “family violence” OR “community violence” | “I think sexual abuse is something that causes a great deal of trouble” [P51] |
| Multiple trauma/recurrent, chronic exposure | If participant described the cumulative nature of many exposures | “In this particular case, there were other situations of violence” [P10] |
| **Mental health consequences of violence** | | |
| Self-harm and suicide | If self-harm or suicide (ideation, behavior, completed suicide) is described | “Had a suicide attempt” [P6] |
| PTSD-like symptoms | If the following symptoms are described:   - Flashbacks, intrusive memories, or nightmares - Avoidance (person avoids reminder of the traumatic event) - Hyper-arousal (if the person described feeling on edge) | “She keeps remembering all these things that she went through” [P38] |
| Depression and anxiety | If person describes symptoms of depression (e.g., negative mood, not enjoying doing things, guilt, low self-esteem) or anxiety (panic, phobias, not wanting to stay alone) | “I see more that it generates depression, it generates anxiety, it generates fear” [P11] |
| Sleep disturbances | If person describes sleep disturbances (e.g., not managing to sleep) | “She [kept] going on telling and how much this had impacts and damages on her life, on her school life, of not being able to sleep” [P27] |
| Substance misuse | If person describes misusing a substance or addiction issues (of any substance) | “I can tell you that there are 60% of my users that I refer to have suffered domestic violence, over 60%, and sexual violence at least 60%. This continues throughout the process and manifests in various aspects of life, including in the increase in alcohol and drug use” [P22] |
| Behavioural and relationship problems | If person describes issues concerning antisocial behavior or aggressiveness, difficulties interacting with others | “It's a child who is more aggressive” [P3] |
| Other mental health symptoms | Any other mental health issues not covered in the categories above (e.g., obsessive compulsive disorder, autism, schizophrenia, bipolar disorder, personality disorders, psychosomatic complaints) | “She starts to sweat, to get tachycardia” [P38] |
| Resilience | If participant speaks of resilience | “The diagnosis I've already made, it's not common. This will depend a lot on the resilience of each one” [P37] |
| Impact on functioning | How did mental health problems in the aftermath of trauma impact functioning (e.g., social, occupational)? | “It is a child who is more aggressive, his performance at school has worsened” [P3] |
| Impact of child/adolescent trauma on adult mental health | If participant notes the impact that trauma during childhood/adolescence can have on mental health in adulthood | But when violence is very embedded in the family relation, it is very rooted [calcada], it is very hidden, we don't notice it, I think we will only see [violence] later on, when they are already adults” [P44] |
| Impact of trauma on larger family system | If participant describes how exposure to trauma in one individual has ripple effects on larger family group | “Often the teenager's story is the story of the house” [P44] |
| **Response, coping, treatment** | | |
| Habituation and/or normalization to violence | If participant mentions that they think people get used to or get habituated to trauma, or that violence is normalized | “Or maybe there is a normalization too. Then they watch things that would probably leave them traumatized, and they're not even scared” [P24] |
| Perceptions about the families and their participation | If participants mention that families can help or harm in the adolescent's mental health care  If participants describe how mental health of parents/caregivers impacts child/adolescent mental health | “Having the search of the users' own families for this mental health care” [P1] |
| Accessing treatment/help seeking | What is the process through which people accessed treatment?  How do people seek help for treatment | “She ended up telling an aunt, and the aunt went there to ask [for support]” [P43] |
| Coping | What are the strategies that adolescents were using to cope with their mental health problems because of trauma? [as reported by provider] | “They only move within the territory where they feel most protected” [P29] |
| Response | What treatment/response was offered to people presenting with mental health problems following trauma? | “Then I sent her to CAPS, she is being medicated, she is taking antipsychotic” [P23] |
| **Clinical considerations on PTSD/CPTSD and adolescent mental health** | | |
| Diagnosis | How is the process of making a diagnosis of PTSD/CPTSD described?  How is the concept of a diagnosis described? (including critical perspectives on diagnosis) | “We have other diagnoses, we work, of course, with nursing diagnoses that are focused on the process of caring for the person and the community” [P33] |
| Critical perspectives of trauma/PTSD | If the respondent provides a critical perspective of the construct of trauma or PTSD, social determinants more important than trauma, importance of context | “The problem is not just post-traumatic stress disorder, but the whole environment. It is not enough for us to treat PTSD; we also have to make the other approaches and other appropriate referrals" [P49] |
| Perceptions of commonality or lack of commonality | If the respondents mentioned how common they think PTSD is in their patient population, how commonly it is discussed within clinical setting etc. | “No, it’s not common” [P46] |
| Perceptions of appropriateness | If the respondents describes that they think PTSD/CPTSD are appropriate for the context, capture the symptom presentation well | “And for him I think, we ended up, I ended up considering that the most adequate diagnosis for him was a Complex PTSD” [P7] |
| Perception of appropriateness/commonality/value/usefulness of CPTSD specifically | If participant describes how they feel CPTSD is particularly useful, appropriate, common in the context of ongoing, chronic, and repeated violence | “And for him I think, we ended up, I ended up considering that the most adequate diagnosis for him was a Complex PTSD” [P7] |
| Lack of precise knowledge | If participant mentions how they are not very sure of their answers concerning PTSD/ they feel they don’t know enough about the topic/ feel like they do not have expertise/feel like they need more training on this | “I have never seen any user with this diagnosis, but I know by hearing about it” [P1] |
| Misdiagnosis | If participant describes issues around misdiagnosing PTSD/CPTSD 🡪 giving the wrong diagnosis | “It [PTSD] ends up being fitted into a little house of anxiety and depression" [P38] |
| Comorbidity | If participants mentions that PTSD is usually together with another psychiatric diagnosis | “When we only have Post Traumatic Stress Disorder, it is easier, but generally the cases that are here at CAPS have a combo, usually it is not just Post Traumatic Stress” [P12] |
| Struggling to remember real example | If participant says they struggle to remember or identify a real example | “And then I'm thinking, if I think about it, it would be like this, I could have other examples, now I can't bring you right away” [P6] |
| Invisibility of adolescents | Quotes that express lack of public policies,  lack of training for professionals related to the subject of adolescent mental health. | “I think there is a difficulty in carrying out this mental health care for these adolescents who are victims of violence, and consequently this diagnosis [PTSD] does not come, because the support there, thinking about health, is not offered thinking about the violence that the adolescent suffers” [P1] |
| **Other** | | |
| Age groups | If the respondent mentions the impact of trauma/PTSD in specific age groups  If participant mentions that they think PTSD/PCTSD presents differently in different age groups | “I think so, maybe more in CAPS Infantil, which picks up the beginning of the story. Because they come here when they're adults, and when they're adults, it's much harder to articulate the family. They only come when they are giving a lot of work” [P2] |
| Impact of COVID-19 on trauma/mental health | If participant describes something related to COVID-19 and pandemic and how that might impact trauma exposure and/or mental health | “I don't know if the pandemic made us look more at this, but diagnosis has been a more present theme” [P21] |
| Poverty, structural inequality | When participants describe wider structural issues such as poverty, racism, discrimination etc. | “I also think that we are in such a precarious territory that we also end up normalizing the different situations of violence. And then community violence ends up being normalized” [P4] |
| Social medicine | When participants use terminology from social medicine (e.g., social suffering, social determinants, and others) | “The problem is not just post-traumatic stress disorder, but the whole environment. It is not enough for us to treat PTSD; we also have to make the other approaches and other appropriate referrals" [P49] |

**Appendix 4**

Mental health consequences of trauma and community violence as described by local health workers

| **Mental health symptoms** | **Quotes** |
| --- | --- |
| PTSD symptoms | "She started to have a lot of autonomic symptoms, flashbacks, nightmares, she started to avoid going to school” [P6]  “When she was looking for a job, she started having intrusive thoughts, when she was taking a bus, she would experience flashbacks of that” [P23]  “Imagining that even though the father is in the northeast [of Brazil] that at any moment he could appear at her door” [P27]  “She keeps remembering all these things that she went through” [P38]  “But the teenager found it very difficult to get in and out of the house, she was very afraid of repeating this movement because she always thought it would happen again” [P40]  “She was afraid of that situation happening again” [P41]  “When she tried to sleep, she kept reliving those situations and everything” [P47] |
| Anxiety and depression symptoms | “The teenager who was... Who was caught by the police committing a theft, and at the Casa Foundation [youth justice center], I think he went through violence there. And now he's locked up at home in a considerable degree of depression” [P4]  “She had a lot of anxious crises at school, and she started to get very depressed too” [P6]  “I see more that it generates depression, it generates anxiety, it generates fear” [P11]  “Develops anxiety issues, depression issues” [P13]  “Adolescents who are vulnerable in the territory, after being threatened too, begin to suffer from cases of anxiety, more phobic” [P16]  “This has caused more anxiety, or more depression, as community violence has been affecting the individual body as well” [P21]  “But I think that in general what we see, I think that the most common symptoms are depression and anxiety” [P27]  “She ended up with a major depression” [P35]  “Then I started to see that they were more symptoms of anxiety” [P41]  “It was the case of a teenager, she was around 14 years old, had suffered sexual violence by a family member and then she really started to develop symptoms of anxiety and fear, of panic disorder” [P43]  “She arrived at us with a picture of really very intense anxiety” [P47]  “And she had a lot of issues, panic syndrome” [P49] |
| Self-harm and suicide | “We have children in this case, I don't know if it characterizes as post-traumatic stress disorder, but we see teenagers who suffer bullying at school and who come to the CAPS more depressed, and then with low self-esteem, with suicidal ideation, the, that was triggered was bullying” [P3]  “She had a series of suicide attempts” [P6]  “Because they have thoughts of ending their own life with so much anxiety” [P23]  “And then the answer to this frequent aggression was to drop out of school because they were attacked in a certain way until they gave up the scholarship and this issue of self-mutilation too” [P28]  “So, during her pregnancy she had a series of suicide attempts” [P28]  “There is a case of a girl who suffered sexual violence and ended up having suicidal ideation, she didn't tell her family, she didn't tell anyone, and we only found out when she started having this suicide attempt” [P35]  “If the patient is self-mutilating, and this is related to an event” [P46]  “And then she had this whole issue of the trigger of all the affective memories she was bringing in relation to this, culminating in her trying to self-exterminate [kill herself]” [P48] |
| Substance misuse | “I think that in addition to detecting substance use, we also detected a situation of violence at home with the mother, stepfather, we understood that the teenager did not sleep well, reacted with a lot of violence to touch, affection, reacted in a way very reactive, she was afraid of things that were common, and then we started to get into the story of how she was being treated, what her life was like at home, in addition to the use of the substance” [P17]  “I can tell you that there are 60% of my users that I refer to have suffered domestic violence, over 60%, and sexual violence at least 60%. This continues throughout the process and manifests in various aspects of life, including in the increase in alcohol and drug use” [P22]  “Today she is I think XX years old, but talking to her mother, she told me that she got married at XX [years old], and before that she suffered sexual violence from an uncle, it seems, a godfather. And at the age of XX, he developed alcoholism, drug abuse, and actually this change in affect regulation” [P24] |
| Behavioral and relationship problems | “It's a child who is more aggressive” [P3]  “But he had a lot of risky behavior, self-harm, fugue states [fuga], he complained of psychotic symptoms, he had a very high demand for care, a lot of difficulty in bonding with others, a lot of self-aggression and little response to drug treatment like this" [P7]  “So, there is a child, for example, who has a history of violence and then the mother manages to leave the house and then he breaks this bond of violence, but he is still a very scared child, who does not trust the environment, who can hardly play, who hardly can be creative” [P12]  “It was a boy who came with destructive behavior, very aggressive, very impulsive” [P12]  “There is a recent teenager that I took in, and then, in discussion with a doctor, we believe that she is very consistent with the diagnosis of PTSD. She is an adolescent who suffered a situation of violence, she cannot say in which space, but she is a more isolated adolescent with great difficulty in having a relationship with other people, including with professionals there, because of this history of violence. She developed anxiety issues, depression issues, and life and death issues as well” [P13]  “She has a lot of difficulty in having relationship with people, more long-term relationships, so her affections are very oscillating and at all times she has a feeling of guilt she has, of self-deprecation” [P13]  Because it is from this history of chronic violence that he had at home that he was like this. Anxious, with terrible self-esteem. Then this week he was saying that he is starting to lose his relationship, because of this insecurity, of thinking that he doesn't deserve the other's love. So, I think it [CPTSD] applies. This is just an example, but I think it [CPTSD] applies” [P16]  “Reacted with a lot of violence to touch, affection, reacted in a way very reactive” [P17]  “Yes, the specific case I think was a matter of physical violence and the child started with a sleep disorder, and started with aggressiveness at school, sleep disorder, he went back to bed-wetting, bedwetting, he was already XX years old. And they are, finally, symptoms of post-traumatic stress” [P46] |
| Sleep disorder symptoms | “So, she started every night, when she went to sleep, she couldn't, because she was having flashes of when she was abused or when she was assaulted” [P23]  “And then after that she couldn't sleep, so she went to sleep in her grandmother's bed” [P24]  “She [keeps] going on telling and how much this had impacts and damages on her life, on her school life, of not being able to sleep” [P27]  “The child started with a sleep disorder” [P46]  “There was a... she was a teenager, she was XX years old, she had suffered more than one robbery within her work. She came to us with really very intense anxiety, insomnia, when she tried to sleep, she kept reliving those situations and everything else, then she told us that she had suffered three consecutive armed robberies like this while at work” [P47] |
| Other (e.g., psychosomatic complaints, psychosis etc.) | “She starts to sweat, to get tachycardia” [P38]  “He complained of psychotic symptoms” [P7]  “And then what comes out is that this boy was exposed for a long time to situations of domestic violence. The parents separated and this violence continued at the grandmother's house. And he became more and more obsessive. More obsessive, and today he finds it difficult, for example, to leave the light on because he doesn't want to spend, to turn off his cell phone because he doesn't want to spend” [P16]  “It's a teenager that we received, it's not from my mini team, it was during the infirmary, he didn't use substances and he first developed a cognitive deficit, they were actually investigating, they hadn't made the diagnosis of autism yet. But he already had a cognitive deficit, he was very introspective and then when we got in touch with a family member, we found out that he had suffered sexual abuse since he was little by an aunt's stepson, I think it was his cousin's aunt, something like that a family member. And he was abused from a young age over and over again and his mother physically assaulted him, so it had repercussions, he was a super blunt/apathetic [embotado] teenager, very puerile, when he talked to us it seemed like a 5-year-old child” [P19]  “In my other job, I had already received an adolescent diagnosed with PTSD at the violence follow-up service, but after we started to follow up, it was actually psychosis, in both cases. There may even have been PTSD at the beginning, but what happened when it came to us was already much more psychotic than anything else, then finally it was diagnosed that it was actually psychosis in both cases” [P29]  “Because she actually came because of a complaint of tremors, her hands were sweating, she couldn't get out by herself” [P33]  “Any running around, and one is already scared. If anyone is running, it is already a reason to be thinking… if you hear fireworks, you already think it’s a gunshot. Thus, there is a high level of stress like that. We receive adolescents who were born there in the community, who were raised there all the time, they even develop schizophrenia, thinking that they are being listened to, that they are being watched, more persecutory [beliefs]. I believe that that is because of the routine [violence] that they end being exposed to” [P40]  “There was a story that she was starting to have a lot of sweating, a lot of tightness in her chest, her heart was beating very hard, and she didn't know why she was feeling like this” [P41] |
